# Supplementary material for: Development and accuracy evaluation of a new loop-mediated isothermal amplification assay targeting the HSP70 gene for the diagnosis of cutaneous leishmaniasis
Source: PLoS One. 2024 Aug 22;19(8):e0306967. doi: 10.1371/journal.pone.0306967 (PMC11340985; doi:10.1371/journal.pone.0306967)

Raw image of a 6% polyacrylamide gel used on fig 1A. Results of the evaluation of different concentrations of the Bst DNA polymerase enzyme and primers used in LAMP-Leish/HSP70 assay. Loading order of samples: 1) molecular weight marker (100bp); 2) PC 4 units *Bst;* 3) NTC 4 units *Bst;* 4) PC 6 units *Bst*; 5) NTC 6 units *Bst;* 6) PC 8 units *Bst;* 7) NTC 8 units *Bst;* 8) PC 8:2 ratio primers; 9) NTC 8:2 ratio primers; 10) PC 16:2 ratio primers; 11) NTC 16:2 ratio primers. Legends:PC – positive control; NTC – negative control; X – lane without sample

1 X 2 3 4 5 6 7 X 8 9 10 11 X X

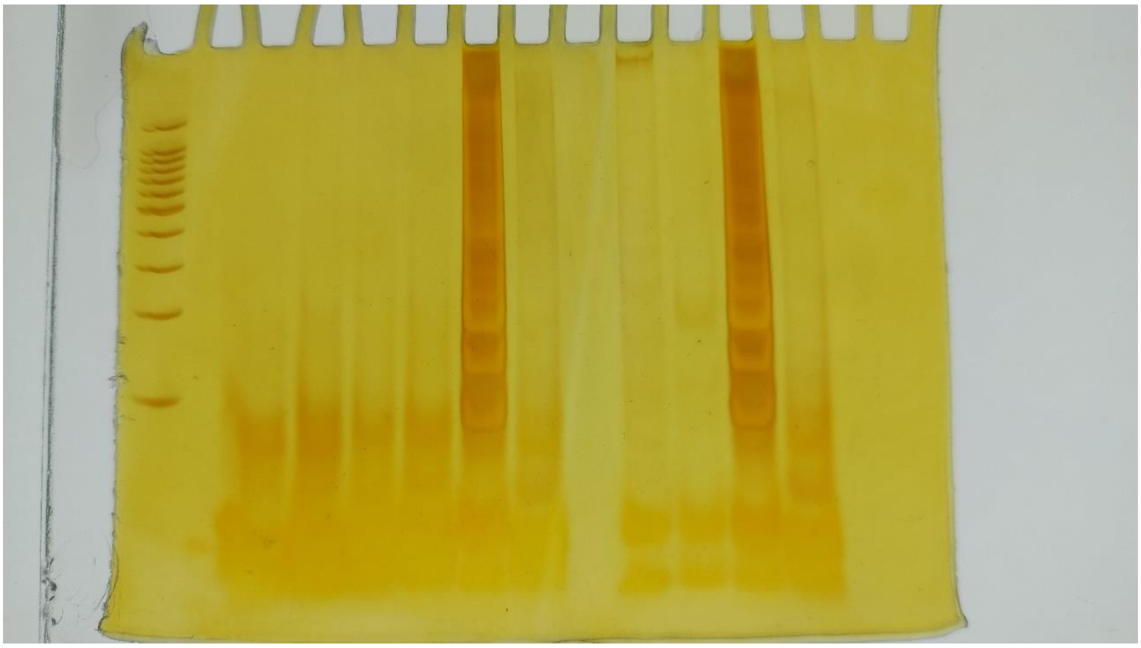

Raw image of a 6% polyacrylamide gel used on fig 1B. Results of the evaluation of different reaction time of LAMP-Leish/HSP70 assay. Loading order of samples: 1) molecular weight marker (100bp); 2) PC 30 min; 3) NTC 30 min; 4) PC 40 min; 5) NTC 40 min; 6) PC 50 min; 7) NTC 50 min; 8) PC 60 min; 9) NTC 60 min; 10) PC 90 min; 11) NTC 90 min. Legends: PC – positive control; NTC – negative control; X – lane without sample

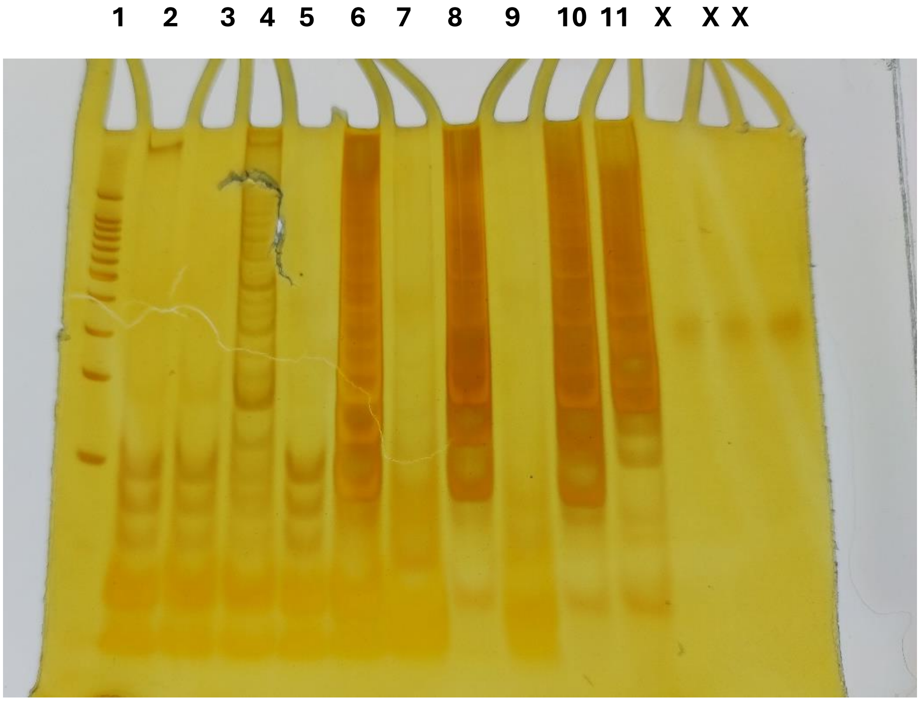

Raw image of a 6% polyacrylamide gel used on fig 2 . Results of the detection limit of LAMP-Leish/HSP70 assay. Loading order of samples: 1) molecular weight marker (100bp); 2) 1 ng; 3) 100 pg; 4) 10 pg; 5) 1 pg; 6) 100 fg; 7) 10 fg; 8) 1 fg; 9) NTC. Legends: NTC – negative control; X – lane without sample

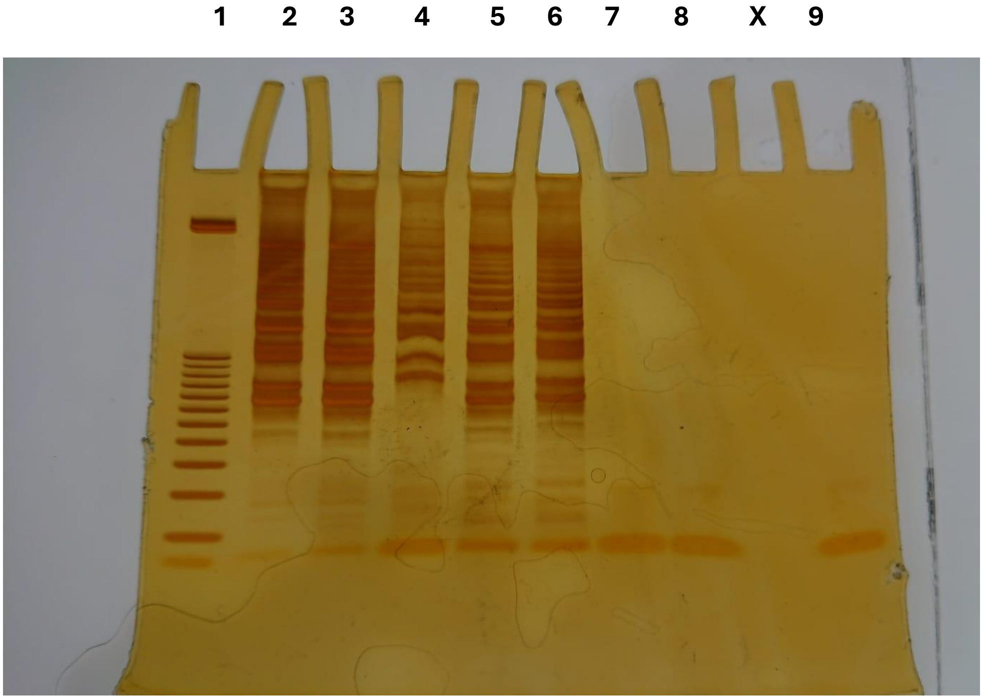

Supplement: S1 Raw images — (PDF) [file pone.0306967.s002.pdf]
