# Supplementary material for: Development and accuracy evaluation of a new loop-mediated isothermal amplification assay targeting the HSP70 gene for the diagnosis of cutaneous leishmaniasis
Source: PLoS One. 2024 Aug 22;19(8):e0306967. doi: 10.1371/journal.pone.0306967 (PMC11340985; doi:10.1371/journal.pone.0306967)
Supplement: Attachment — Submitted filename: Review.docx [file pone.0306967.s003.docx]

The submitted paper is well driven and composed. However, there are some incongruences that need clarification.

Comments:

-Line 27: what species do you want to amplify?, CL is caused by several species and that is important for the specificity and the design of the LAMP assay. Some epidemiology data is lacked here. This would explain why you use conserved regions for the design (line 101).

-Line 67: indicate the clinical sensitivity of CL Detect™ Rapid Test for comparison.

-Line 70: It is lack of data regarding sensibility and specificity of molecular techniques such as PCR. Please, describe it. It is important to know the accuracy of molecular data – this is why a LAMP amplification is pursued in this work.

-Line 83: why did you design a new LAMP assay instead of using a previously standardized LAMP technique?

-Line 93: highlight the advantages of choosing these targets for this new LAMP assay.

-Line 94: I would mention that you tried to standardize 18S-LAMP assay without success and remove this assay from the draft due that there are other protocols already working good (León et al, 2018; Ghodrati et al, 2017; Tiwananthagorn et al., 2017; etc..). There is no point in its description, once you have not used it for diagnosis, main objective of the manuscript.

-Line 281: what are the sensitivity rates presented by the reference q-PCR and direct microscopy examination and aspirate of culture?, similar to LAMP?, introduce this information in Table 3.

-Line 292: data from q-PCR are lacking. What was the cut-off (line 186)?. Where those negatives by LAMP (FN) the ones with lower parasitic load by kDNA-qPCR?, is a problem of sensibility?. Do you know the specie causing the biopsies?, is it a problem of specificity regarding some Leishmania species?

-Line 297: why did you use 10 parasites/µg of DNA tissue?.

Please for comparison describe accuracy of all the cited references in Discussion.

-Line 319: it is described the LOD of purified Leishmania DNA which is different from Leishmania DNA in biopsies (they include human DNA). Please verify if the LOD is from purified DNA or biopsies in the references added, and insert a range of LOD from these references in the text.

-Line 334: insert the performance of the cited references in the text as a range.

-Line 336: why is this described apart regarding line 334?, My advice is to cite here only those with PCR as a reference (microscopy results has a poorer relationship). This will help to compare with the protocol optimized here. Are there any study including a PCR protocol using a HSP70 target?.

-Line 341: please describe the results of these studies for comparison. Other places out of Latin American countries?, which is the target? is a conserved target among species?.

-Line 351: describe target, specie..

-Line 354: any PCR, correct it.

-Line 355: what is the advantage regarding the other LAMP protocols?

-Line 357: 357: as other LAMP protocols?

-Line 361: did your LAMP protocol suffer from inhibition?, did you add SYBR green to avoid it?, all LAMP protocols that not use SYBR green are prompt to be inhibited? Because you have cited several studies in your discussion with good results.

-Line 374: why did it appear here mucosal leishmaniasis?, not described before its importance. If so, why did not do it in your study?

COMMENTS TO THE EDITOR:

The authors seem to have a consistent experience in the field. However, some improvements should be done and some recommendations have been included to the authors. From my point of view, main limitations of the work are: a) lack of description of the accuracy of other techniques cited in the text (for comparison); b) lack of description of the advantages of this technique regarding others already published or manufactured; c) 18S-LAMP assay should be removed from the manuscript.
