# Supplementary material for: Development and accuracy evaluation of a new loop-mediated isothermal amplification assay targeting the HSP70 gene for the diagnosis of cutaneous leishmaniasis
Source: PLoS One. 2024 Aug 22;19(8):e0306967. doi: 10.1371/journal.pone.0306967 (PMC11340985; doi:10.1371/journal.pone.0306967)
Supplement: Attachment — Submitted filename: ResponseReviewers_PlosOne_version2.docx [file pone.0306967.s005.docx]

We would like to thank the academic editor and reviewers for their careful review of the manuscript **"Development and accuracy evaluation of a new loop-mediated isothermal amplification assay targeting the HSP70 gene for the diagnosis of cutaneous leishmaniasis"** (PONE-D-24-03257).

**Answer Letter to Reviewers**

Reviewer #1:

• Line 47: remove “as”

Done.
• Line 67: indicate the clinical sensitivity of CL Detect™ Rapid Test for comparison.
Rephrase the sentence in line 65: “been evaluated in some endemic countries (31.3%-65.4% sensitivity when compared to microscopy and PCR) [12-14].

This information was added.
• Line 82: delete “.” after respectively

Done.
• Line 84: The previous 18S cross-reactivity with Trypanosoma cruzi DNA should be mentioned (de Faria et al., 2022).

This information was added, as mentioned in reference 28.
• Line 92: highlight in a sentence the advantages of choosing HSP70 for this new LAMP assay.
- most common target used for Leishmania species identification,
- presents conserved and polymorphic regions: being able to differentiate a wide range of species from different geographical origins, especially the ATL species,
- Etc

Sentence was rewritten with the information added. Lines: 96-99.
• Line 93: Rephrase the sentence:
The target was selected because is commonly used for molecular diagnosis of Leishmaniasis, including Leishmania species identification. Besides that, this is the first LAMP assay based on HSP70 gene for DNA detection of Leishmania. The accuracy of LAMP- Leish/HSP70 assay was evaluated for the diagnosis of CL.
The target was selected because is commonly used for molecular diagnosis of Leishmaniasis, including Leishmania species identification. For the first time, the accuracy of LAMP- Leish/HSP70 assay to diagnose CL was evaluated in this study.

Sentence was rewritten with the information added. Lines: 96-99.
• Line 167: The recruitment period occurred from 2017 to 2019.

Done.
• Line 307: : delete “)” here e;f)

Done.
• Line 310-12: Please indicate where these data are in the paper. If not, add median values and IQR to the text:

Recent lesions (median of 90 days) had a significantly higher parasite load compared to lesions with a longer evolution time (median of 120 days) (U Mann-Whitney test, p = 0.03).

The values were added, and analysis was remade. There was a typing mistake in the p value (it was 0.03 and the correct one is 0.3), that was corrected and the interpretation was rewritten. The raw data was referenced. Lines: 315-320.
• Discussion: Each of the available PoC diagnostic methods has a limitation in at least one REASSURED criterion. It is very difficult to create pathogen-specific PoC diagnostic platforms that meet all of these criteria. In limitations, the authors should cite that Loop-mediated isothermal amplification (LAMP) is a rapid, user friendly isothermal nucleic acid amplification method that has almost all ASSURED properties. They might include some information from <https://doi.org/10.1080/14737159.2021.1873769>.

Thanks for the suggestion. This information and the reference were added. Lines: 404-409.
• Write consistently one of each term along the text: hsp70-LAMP assay or HSP70-LAMP assay or LAMP-Leish/HSP70 assay

Done. We kept “LAMP-Leish/HSP70 assay” in the text.
• Figure 1. Please, improve the typewriting, it is unreadable

The typewriting is unreadable due to compression of the file made available in the PLOS editorial manager platform. The uncompressed version of all images, uploaded following PLOS guidelines, are present in the editorial manager, under "File Inventory" link.

**Reviewer #2:**

- Lines 169-171: This sentence must be removed: “All individuals participated voluntarily in the study and informed consent was obtained from each participant of from their legal guardian for underage participants”, because it repeats information already stated in lines 165-167.

The sentence was removed.

- Table 1, first line, change “pb” into “bp” to be standardized with the legend.

Done.

- Line 363: “(…) in the inner side of the tube ever (…)” must be replaced by: “(…) in the inner side of the tube cover (…)”

Done.

- Line 420: “This study was supported by and The Research Foundation (…)” must be replaced by: “This study was supported by The Research Foundation (…)”

Done.

- Reference 16 must be adapted to Vancouver style.

Reference was adapted to Vancouver style.
